# Label-Free Three-Dimensional Morphological Characterization of Cell Death Using Holographic Tomography

**DOI:** 10.3390/s24113435

**Published:** 2024-05-26

**Authors:** Chung-Hsuan Huang, Yun-Ju Lai, Li-Nian Chen, Yu-Hsuan Hung, Han-Yen Tu, Chau-Jern Cheng

**Affiliations:** 1 Institute of Electro-Optical Engineering, National Taiwan Normal University, Taipei 11677, Taiwan; terry.chhuang@gmail.com; 2 Department of Life Science, National Taiwan Normal University, Taipei 11677, Taiwan; 60843038s@gapps.ntnu.edu.tw (L.-N.C.); 61043037s@gapps.ntnu.edu.tw (Y.-H.H.); 3 Department of Electrical Engineering, Chinese Culture University, Taipei 11114, Taiwan; dhy@ulive.pccu.edu.tw

**Keywords:** holographic tomography, three-dimensional morphology, cell death

## Abstract

This study presents a novel label-free approach for characterizing cell death states, eliminating the need for complex molecular labeling that may yield artificial or ambiguous results due to technical limitations in microscope resolution. The proposed holographic tomography technique offers a label-free avenue for capturing precise three-dimensional (3D) refractive index morphologies of cells and directly analyzing cellular parameters like area, height, volume, and nucleus/cytoplasm ratio within the 3D cellular model. We showcase holographic tomography results illustrating various cell death types and elucidate distinctive refractive index correlations with specific cell morphologies complemented by biochemical assays to verify cell death states. These findings hold promise for advancing *in situ* single cell state identification and diagnosis applications.

## 1. Introduction

Cell death is a fundamental process in cell biology [1,2,3] and holds significant importance. Besides traditional types like apoptosis [4] and necrosis [5], several other types of cell death have been reported. The classification of cell death now includes non-programmed necrosis [6], programmed apoptosis, and programmed non-apoptotic cell death, which encompasses autophagy [7], mitoptosis [8], pyroptosis [9], and ferroptosis [10]. Since these newly observed cell deaths have distinct mechanisms and phenotypes, the morphological changes between them are also distinct [11]. Apoptosis causes fragmentation of the cell into small apoptotic bodies, while necrosis is characterized by cell swelling and rupture, leading to the release of cellular contents that can trigger inflammation and tissue damage [12]. Furthermore, necroptotic cells can exhibit swelling and the formation of blebs on the plasma membrane. The cytoplasm can also become densely packed, and organelles such as mitochondria can become swollen or disrupted [13]. In some cases, cells can also undergo rupture, leading to the release of cellular contents and an inflammatory response [14]. Autophagic cell death involves the formation of large autophagic vacuoles or autolysosomes within the cell [15]. The cytoplasm can become dense with these structures, and the nucleus can undergo chromatin condensation or fragmentation [16]. In pyroptosis, cells also undergo swelling and the formation of membrane pores that allow the release of pro-inflammatory cytokines. Dense cytoplasm and a fragmented nucleus can also be observed [17]. Ferroptosis, a form of iron-dependent, ROS-induced cell death, is characterized by various morphological changes including cell shrinkage, membrane blebbing, and the formation of lipid droplets [18]. The mitochondria can also become condensed, and the cristae can become disorganized [19]. It is important to note that these morphological changes are not always specific to a particular form of cell death and can overlap with other forms of cell death or cellular stress responses. Therefore, a combination of morphological and molecular criteria is often used to identify and characterize non-apoptotic forms of cell death.

To observe cell morphology, the conventional imaging method involved the combination of a bright-field microscope [20] and a fluorescence microscope [21]. The contrast in the bright-field microscope images was derived from the transmittance of the cells, allowing for the observation of cell appearance and location through differences in transmittance. However, bright-field microscope images only provided a rough representation of cell appearance and could not visualize internal organelles. To visualize cell organelles, specific fluorescent dyes were used to stain the organelles, and fluorescence signals were then excited with specified wavelength bands. Organelles were defined based on these fluorescence signals. However, in most cases, a series of treatments were required before fluorescent imaging could be conducted. The time-consuming nature of these treatment procedures made measuring living cells challenging and could potentially introduce disturbances and side effects to the cells. Therefore, developing imaging instruments capable of measuring cell morphology and internal organelles in a label-free manner was crucial.

Holographic tomography (HT) [22,23,24,25] is a powerful label-free imaging technique that enables the observation and quantitative analysis of cells and their internal organelles in three dimensions (3D) based on refractive index (RI). The RI distribution of a cell is sensitively related to its mass distribution [26], and different organelles exhibit distinct RI distributions. Therefore, RI analysis can provide valuable insights into diverse biological models. Physical parameters of cells and organelles, such as surface area, height, volume, etc., can be calculated based on RI distribution. HT has been employed in numerous studies to measure cell morphology, including imaging cadmium telluride quantum dots-induced cell apoptosis [27], analyzing cell morphological changes in response to photodynamic treatment *in vitro* [28], investigating sarcoma cell elimination by calcium electroporation [29], non-invasive monitoring and evaluation of the antitumor effect of traditional Chinese medicine on 4T1 breast tumor cells [30], and other biological applications [31,32,33].

However, the cell death in different cell lines may be induced by various environmental conditions exhibiting inter-and intra-cell morphology and structure changes during cell death pathways. In this study, we investigate the 3D morphological changes associated with neuroblastoma (SH-SY5Y) cell death induced by autophagy, apoptosis, and ferroptosis using the HT imaging technique. To facilitate comparison, we utilize biochemical assays such as immunofluorescence imaging and western blot analysis [34] to assess cell states. For observing 3D morphological alterations, we employ an HT system to examine cellular and organelle structures. Our experimental findings reveal distinct morphological changes induced by cell death. Specifically, autophagy leads to cell shrinkage and the appearance of small particles with a high refractive index (RI) within cells, while apoptosis triggers cell fragmentation and nucleus condensation into a circular shape. Ferroptosis cells exhibit similarities to the normal state, albeit with a slightly higher nucleo-cytoplasmic ratio (NCR). Quantitative analysis of 3D RI distribution across different cell states reveals changes in projected area, height, section area, volume, and refractive index, enabling determination of cell states. We anticipate that label-free 3D RI imaging coupled with quantitative analysis will facilitate the identification of cell death states.

## 2. Materials and Methods

### 2.1. Cell Culture

SH-SY5Y neuroblastoma cells were cultured in DMEM/F-12 culture medium (Gibco, Emeryville, CA, USA) containing 10% Fetal bovine serum (Sigma-Aldrich, Munich, Germany) and 1% Penicillin/Streptomycin (Gibco, CA). The cells were maintained in a 37 °C incubator supplied with 5% CO_2_.

To induce autophagy, 4 × 10^5^ cells were seeded in 35 mm dishes for overnight attachment and then transfected with GFP-LC3 (microtubule-associated protein 1A/1B-light chain 3) expression plasmid using Lipofectamine 2000 (Invitrogen, Waltham, MA, USA) according to the manufacturer’s instructions. After incubation for 8 h, the medium was changed to serum-free or serum-containing DMEM/F-12 (Gibco, CA) for another 16 h.

To induce cell apoptosis, 4 × 10^5^ cells were seeded in 35 mm dishes (Cellvis, Mountain View, CA, USA) with 5% gelatin-coated cover slips or 60 mm dishes (NEST, Wuxi, China) overnight and then exposed to UV for 1 h followed by a 30-min incubation in the incubator.

To induce ferroptosis, 1.5 × 10^5^ cells were seeded in 35 mm dishes containing 5% gelatin (Sigma-Aldrich, Munich, Germany)-coated cover slips overnight and then changed to DMEM/F-12 culture medium containing 10 mM sodium iodate (Scharlau, Barcelona, Spain) for another 24 h. The apoptotic, autophagic, and ferroptotic cells for holographic tomography analysis were all fixed by 3% formaldehyde (Bionovas, North York, ON, Canada) for 15 min.

### 2.2. Apoptosis Assay (Annexin V Staining) and Flow Cytometry for UV-Exposed Cells

The UV-induced apoptotic cells were stained using the FITC Annexin V Apoptosis Detection Kit I (Becton Dickinson, Franklin Lakes, NJ, USA) according to the manufacturer’s instructions. Briefly, UV-exposed cells in 60 mm dishes were trypsinized and stained with annexin V and propidium iodide (PI) for 15 min, then analyzed by flow cytometry (Flow Cytometer and Cell Sorter, FACS Calibur, Becton Dickinson, NJ). UV-exposed cells seeded on the cover slips were also stained with annexin V and PI with the same kit, and then fixed with 3% formaldehyde for 15 min. The slides were then imaged by confocal microscopy and HT analysis.

### 2.3. Propidium Iodide (PI) Staining for Ferroptotic Cells

After treatment with sodium iodate, cells on the cover slips were fixed with 3% formaldehyde for 15 min and then stained with 50 μg/mL propidium iodide (PI, Sigma-Aldrich, Munich, Germany) for 5 min. Stained cells were then mounted by glycerol mounting solution and analyzed by fluorescent microscope.

### 2.4. Immunofluorescence Imaging for Autophagic Cells

Normal or serum-starved cells transfected with GFP-LC3 were further stained with 1 nM LysoTracker Red DND-99 (Invitrogen, MA, USA) for 1 h to label acidic organelles, including endosome and lysosome and DAPI (4′,6-diamidino-2-phenylindole) stain (BioLegend, San Diego, CA, USA) for 10 min to stain the nucleus. Cells were then kept in phenol red-free DMEM/F-12 medium (Gibco, CA, USA) with 10 mM HEPES (Bionuvas, North York, ON, Canada). The autophagosomes or autolysosomes were observed in live cells by confocal microscopy (ZEISS LSM880, ZEISS, Oberkochen, Germany).

### 2.5. Western Blot Analysis

Apoptotic, autophagic, or ferroptotic cells were lysed by sodium dodecyl sulfate (SDS) lysis buffer (0.5% SDS, Tris-HCl, Alpha Chemistry, Taipei, Taiwan) and samples containing 50 μg protein were analyzed by SDS polyacrylamide gel electrophoresis (SDS-PAGE). After blotting with specific antibodies, the signals were detected by LAS4000 Luminescent Image Analyzer (Cytiva, Amersham, UK). The primary antibodies employed included anti-Caspase 8 (GeneTex, Irvine, CA, USA), anti-LC3, anti-Beclin-1, and anti-p62 antibodies (Proteintech, Rosemont, IL, USA) at a 1:1000 dilution, anti-GPX4 and anti-COX2 (Proteintech, IL) at a 1:3000 dilution, and anti-GAPDH (Proteintech, IL) at a 1:10,000 dilution. Secondary antibodies included Amersham ECL Rabbit IgG HRP-linked whole Antibody (GE Healthcare, Anaheim, CA, USA) at a 1:2500 dilution and mouse IgG-heavy and light chain Antibody (Bethyl Laboratories, Inc., Montgomery, TX, USA) at a 1:5000 dilution. The quantification of the relative expression levels of proteins were analyzed by Image J 1.52a software. The levels of GAPDH served as the internal loading control.

### 2.6. Holographic Tomography (HT) System

**Figure 1:**
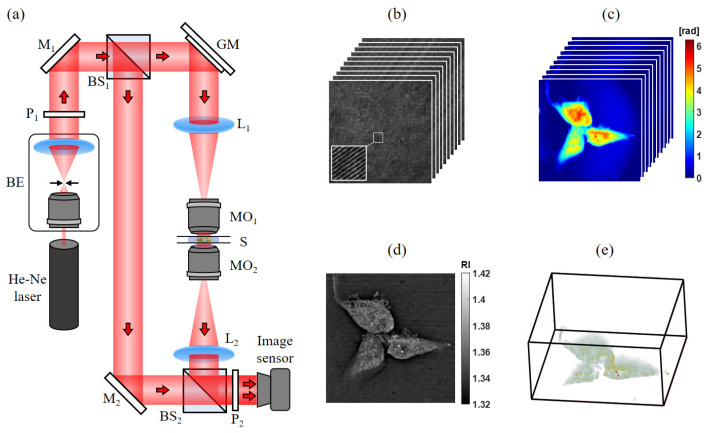
(**a**) Optical setup of HT. The experimental results of (**b**) a series of hologram acquisitions, (**c**) reconstructed phase, (**d**) sectional RI tomogram, and (**e**) 3D RI visualization. BE: beam expander; P: polarizer; M: mirror; BS: beam splitter; GM: galvo mirror; L: lens; MO: microscope objective; S: sample.

The HT system was utilized to measure and analyze changes in 3D cell morphology. The optical setup of the HT system, based on an improved Mach–Zehnder interferometer, is depicted in Figure 1a. A He-Ne laser (Uniphase (St. Charles, IL, USA), 1145P, λ = 632.8 nm, 21 mW) served as the coherence light source in the system. The laser beam was expanded by a beam expander (BE) and split into an object beam and a reference beam by a beam splitter (BS_1_). The object beam scanned the sample at different azimuth angles using dual-axis scanning galvo mirrors (Thorlabs (Newton, NJ, USA), GVS012) and a telecentric lens (L_1_: f = 180 mm; MO_1_: Olympus (Tykyo, Japan), LMPLFLN100x, 100x, NA = 0.8, long working distance). The transmitted wavefront information of the sample at different azimuth angles was collected through another telecentric lens (MO_2_: Olympus, UPLSAPO60XW, 60x, NA = 1.2, water-immersion; L_2_: f = 180 mm) and interfered with a reference beam on the imaging sensor (Qimaging (Surrey, BC, Canada), 0I-ROL-BOLT-M-12, pixel number: 1024 × 1024, pixel size: 3.63 μm) to form digital holograms, as illustrated in Figure 1b. The scanning angle of the object beam ranged from −45° to 45°, resulting in a total of 241 recorded holograms. After recording the digital holograms, they were numerically reconstructed to retrieve the phase distribution of the sample, as shown in Figure 1c. The samples we used were fixed SH-SY5Y cells in different cell death states. The phase information represents the optical thickness of the sample as a function of the illumination angle. Utilizing the HT reconstruction process [35,36,37] using the direct inversion method, the reconstructed object wavefront was Fourier transformed and synthesized into the corresponding azimuth angle in the 3D spectrum. Subsequently, the 3D refractive index (RI) tomogram could be reconstructed by inverse Fourier transforming the synthetic 3D spectra. The RI sectional image and the 3D tomogram are presented in Figure 1d and Figure 1e, respectively. In the HT system, a lateral resolution of up to 200 nm can be achieved (calibrated using a standard spoke target), with a reasonable accuracy of 0.002 in RI change, which is adequate for distinguishing major cellular organelles. When analyzing the microscopic structure of cells, meeting the requirements for lateral resolution and RI changes can lead to higher image contrast, aiding in the recognition of cellular features.

### 2.7. 3D Cell Morphology Segmentation and Analysis

The 3D cell tomogram contains both the cell distribution and the background. To accurately calculate the cell morphology parameters, the cell distribution and background need to be separated using a cell segmentation algorithm. The first step of the cell segmentation algorithm involves separating the cell area from the tomogram based on the RI threshold. Since the background environment is phosphate buffered saline buffer (PBS, RI: 1.334), the RI threshold for cell segmentation is set to 1.34 to create the cell mask. Once the cell mask is preliminarily created, it is optimized through erosion, expansion, and manual selection to accurately separate the cell body from the background area.

To label the nucleus area, the first step involves manually marking the nucleus area in the x-y section image (z = 0), followed by using 3D edge contour detection and ellipse fitting to create a nucleus mask. After labeling the 3D cell body and nucleus area, the cytoplasmic area can be obtained by subtracting the cell nucleus region from the cell body region. Quantitative analysis of cells can then be performed using the cell segmentation algorithm.

### 2.8. Quantification Analysis of Cell Morphology

According to the cell segmentation algorithm, the cell body and nucleus can be separated, and the physical parameters of the cells can be quantified. The data of the cytoplasm can be approximately calculated as the data of the whole cell body minus that of the nucleus. We calculated four physical parameters from the 3D RI tomogram, including section area, height, volume, and average RI. To quantitatively calculate these physical parameters, masks were required to separate the cell region from the background. The masks were generated based on the RI distribution. The cell segmentation processing provided direct calculation of these physical parameters of cell morphology. The detailed definition of these parameters is as follows:

1. The section area refers to the cell distribution at z = 0.
2. The height refers to the maximum height of cells.
3. The volume refers to the 3D distribution of cells.
4. The average RI refers to the average RI distribution of the 3D cell region.
5. The NCR is the ratio of the size (i.e., section area, height, volume, and average RI) of the nucleus of a cell to the size of the cytoplasm of that cell.

The total calculated physical parameters was 16. The number of cells analyzed for calculation per state was approximately 150.

## 3. Results and Discussion

### 3.1. Observation and Verification of Cell Death

**Figure 2:**
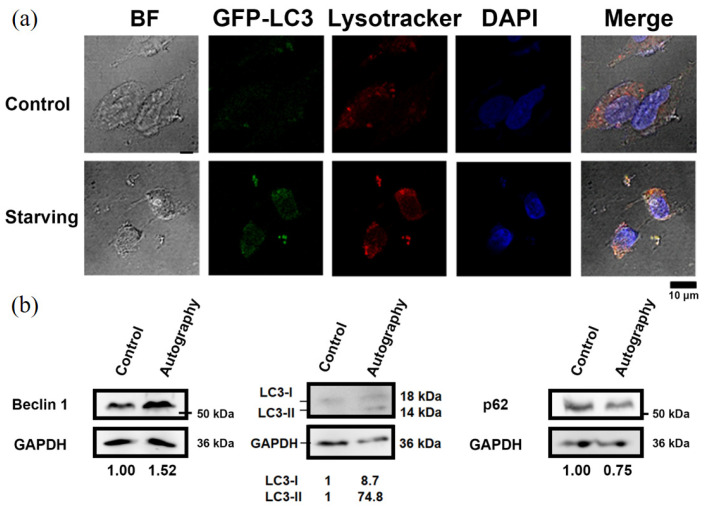
Serum starvation-induced autophagic SH-SY5Y cells. (**a**) Immunofluorescent images for bright field (BF), nucleus staining (DAPI), GFP-fused LC3 protein, and lysosome labeling (lysotracker) of cells. (**b**) Western blot analysis for Beclin-1, LC3, and p62 expression. The relative expression levels of proteins were quantified using Image J software. GAPDH levels were used as the internal loading control.

**Figure 3:**
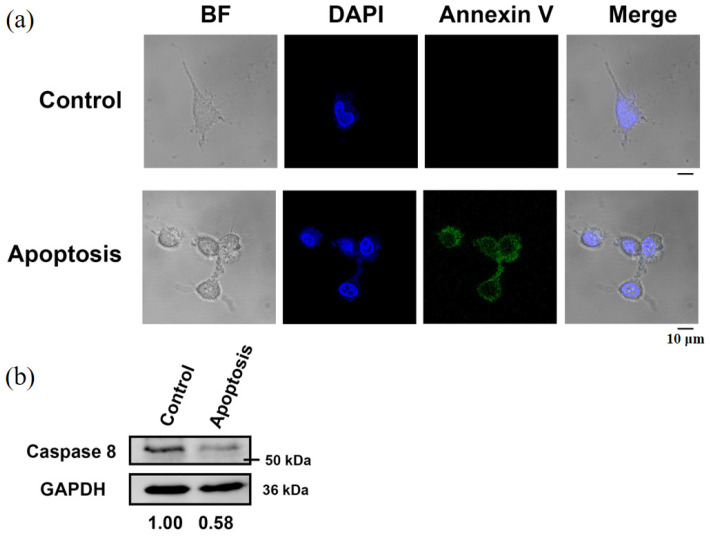
UV-induced apoptotic SH-SY5Y cells. (**a**) Immunofluorescent images for bright field (BF), nucleus staining (DAPI) and annexin V labeling of cells. (**b**) Western blot analysis for caspase 8 expression. The relative expression levels of proteins were quantified using Image J software. GAPDH levels were used as the internal loading control.

**Figure 4:**
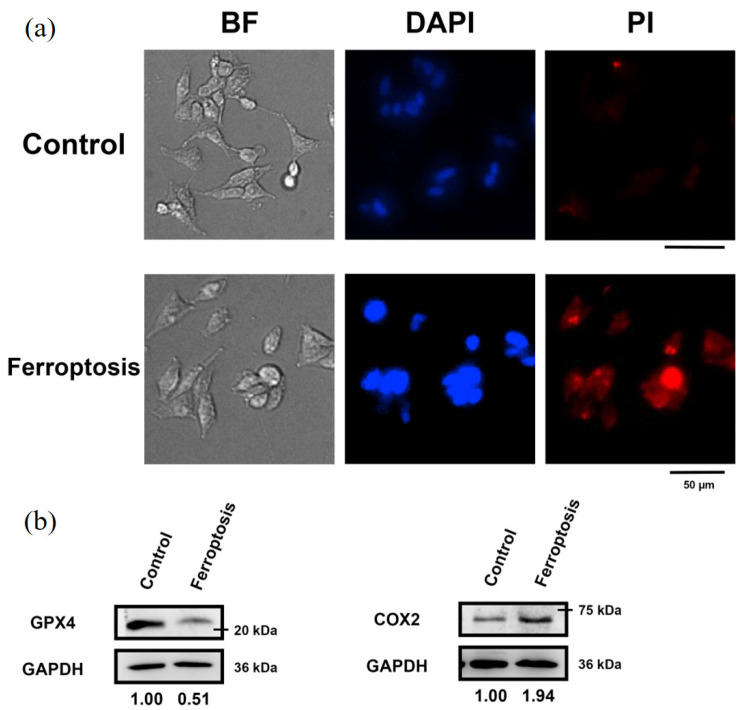
Sodium iodate-induced ferroptotic SH-SY5Y cells. (**a**) Immunofluorescent images for bright field (BF), nucleus staining (DAPI), and propidium iodide staining of cells. (**b**) Western blot analysis for GPX4 and COX2 expression. The relative expression levels of proteins were quantified using Image J software. GAPDH levels were used as the internal loading control.

To prepare dying cells through different programmed death pathways for HT analysis, the cells were initially examined for autophagy (Figure 2), apoptosis (Figure 3), and ferroptosis (Figure 4). Autophagic cells exhibited increased punctate LC3 staining and acidic vesicle staining (Figure 2a). Western blot analysis of autophagy markers also demonstrated increased LC3-II and Beclin-1 expression and decreased levels of p62 (Figure 2b). The cells undergoing different death pathways were confirmed by immunostaining and western blot analysis of specific cellular markers. Apoptotic cells induced by UV radiation were positively stained for annexin V, and the expression levels of pro-caspase 8 were reduced (Figure 3). In ferroptotic cells induced by sodium iodate treatment, dead cells were positively stained with propidium iodide (Figure 4a). Ferroptosis indicators, including decreased GPX45 and increased COX2 expression, were also observed (Figure 4b).

### 3.2. Cell Measurements

**Figure 5:**
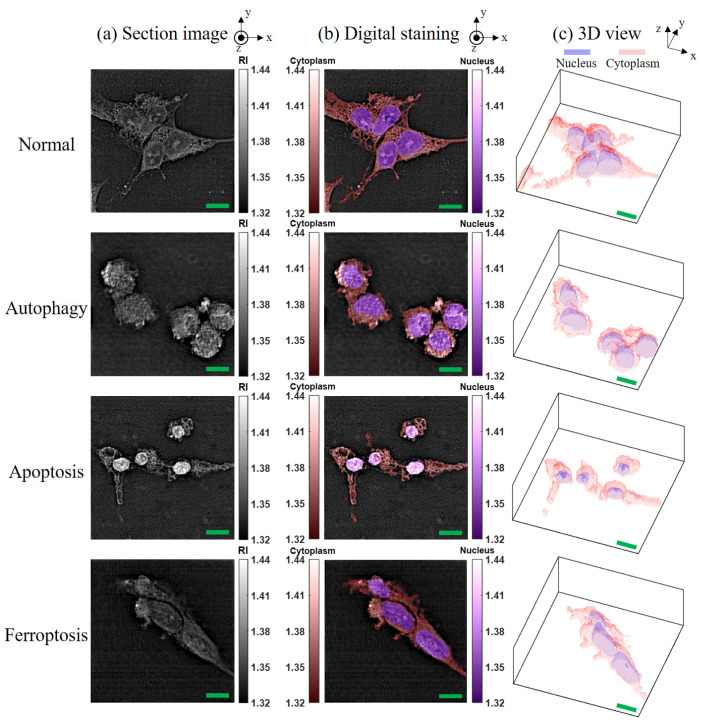
Representative RI image in the SH-SY5Y cell conditions of normal, autophagy, apoptosis, and ferroptosis. (**a**) Section images. (**b**) Digital staining. (**c**) 3D view of cell images. Scale bar (green): 10 μm.

To compare the distinct image properties of different cell deaths using HT, fixed SH-SY5Y cells under various conditions (normal, autophagy, apoptosis, and ferroptosis) were measured and analyzed through the HT system. The image results under different conditions are depicted in Figure 5, which include the original section image of the x-y view at z = 0 (Figure 5a), the digital staining for the section image (Figure 5b), and their 3D views (Figure 5c). As shown in Figure 5b, two colors represent the RI distribution of the nucleus and cytoplasm, respectively, while the gray level denotes the RI values. For the 3D view in Figure 5c, the cell morphology was plotted only in surface mapping for better visualization.

**Figure 6:**
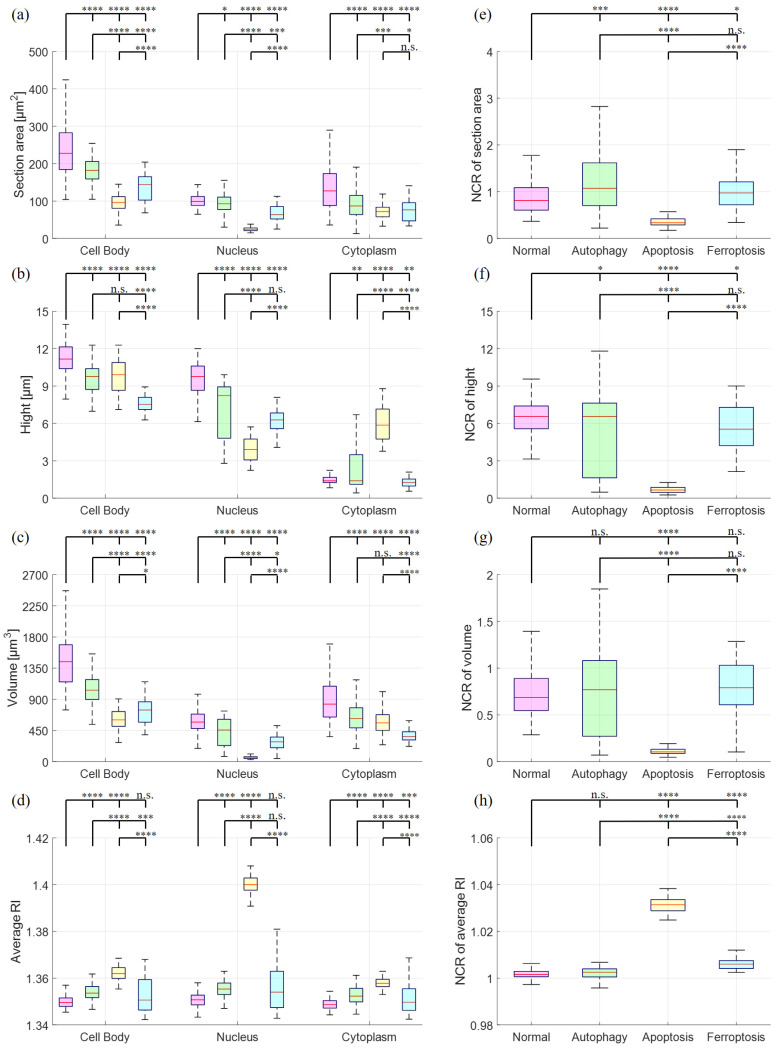
Box plot for quantitative analysis of physical parameters in different cell states. Red: normal cells; green: autophagic cells; yellow: apoptotic cells; blue: ferroptotic cells. The area, height, volume, and average RI were compared in different cell conditions (**a**–**d**), respectively. The NCR of these parameters were also shown in (**e**–**h**), as indicated. n.s.: not significant; *: *p* < 0.05; **: *p* < 0.01; ***: *p* < 0.001; ****: *p* < 0.0001.

**Figure 7:**
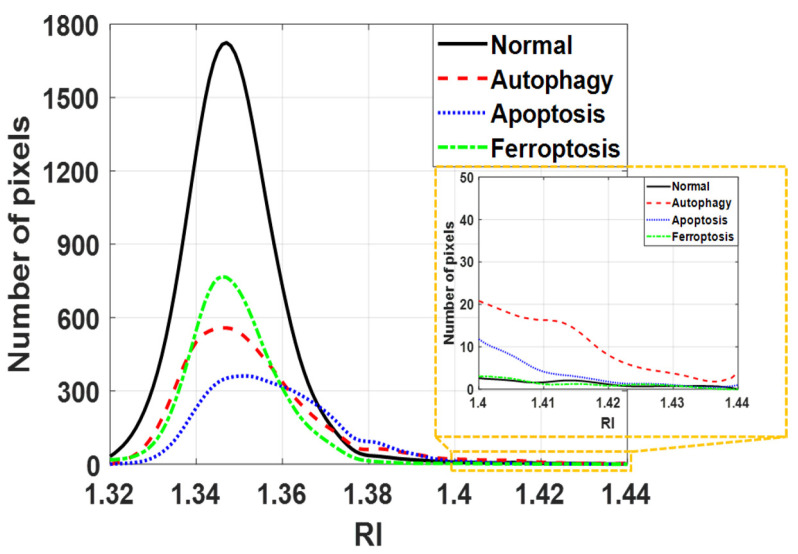
Directly fitting plots of RI histograms calculated from the section images (z = 0) in different cell states. There are relatively high refractive index values in autophagy and apoptosis.

Based on the results of HT imaging and the cell segmentation algorithm, the physical parameters of the cells under different cell states were analyzed in Figure 6. For normal cells, the nucleus and its inner nucleolus were observable, and cell protrusions extended normally. Autophagic cells, induced by nutrient deprivation, exhibited reduced cell volume and decreased protrusion contraction. These results are consistent with the fluorescence image observations (Figure 2). Higher RI areas (RI > 1.42) were observed within these cells, with sizes ranging from 0.8 μm to 1 μm (Figure 7), presumed to be specific structures present only during autophagy. In apoptotic cells, cell bodies were ruptured due to UV light irradiation, and the nucleus shrank into a circular shape, with a size around 7 μm. These results are consistent with the fluorescence image observations (Figure 3). The RI of the nucleus (RI > 1.41) was significantly higher than that of the cytoplasm, as illustrated in Figure 6. In ferroptotic cells, the morphology is similar to that of normal cells which can be observed in the fluorescence image (Figure 4). The difference between the normal cells and ferroptotic cells can only be distinguished through fluorescence images. Through HT imaging, the morphological analysis between the two is also similar, but it can be observed that there are differences in the NCR of the average RI in the ferroptosis state, which was slightly higher than in the normal state.

We also consider the possible effect of cell fixation on the cell morphology characterization of death states. The fixation procedure can influence cell morphology, causing shrinkage/expansion, changes in composition, and potential artifacts [38,39,40]. By selecting the appropriate fixative solution, the issue of cell deformation can be mitigated, thus reducing variations in morphology and biophysical parameters across different cell death states. In this study, a fixative solution comprising 3% formaldehyde, commonly used for cell fixation, was employed to prevent cellular degradation and decomposition during experiments, thereby preserving cellular morphology and structural integrity. Additionally, fixed cells enable repeated measurements, significantly minimizing cell loss.

In summary, the following points regarding biophysical parameters can be highlighted:

1. For autophagic cells, most of the analytic parameter values ranked second highest after normal cells. The highest RI population belonged to the autophagic cells, as illustrated in the directly fitting plots of the RI histograms calculated from the section images in Figure 7. Due to the smaller number of highest RI regions, they were not significantly observed in the average RI value.
2. Apoptotic nuclei exhibited significantly smaller sizes for section area, height, and volume. The RI is significantly increased in apoptotic nuclei but not in other types of cell death, indicating that chromatin/nucleus condensation occurs primarily in apoptosis but not in other types of cell death.
3. Most of the analyzed physical parameters for ferroptotic cells fell between those of autophagic and apoptotic cells.

## 4. Conclusions

In this study, the holographic tomography technique was employed to investigate the morphological changes of apoptotic, autophagic, and ferroptotic SH-SY5Y cells using a 3D cell RI model, complemented by biochemical assays to verify cell death states. Based on the 3D RI tomogram, physical parameters such as section area, height, volume, average RI of cell body, nucleus, cytoplasm, and nucleo-cytoplasmic ratio were analyzed and compared across different cell death pathways. The HT experimental results revealed morphological changes and alterations in physical parameters among different cell death states. Autophagic cells exhibited reduced cell volume and decreased protrusion contraction. Apoptotic cells showed fragmentation of the cell body with the nucleus condensed into a circular shape, as well as the highest average RI value. Ferroptotic cells did not exhibit specific characteristics in the analyzed parameters, with values falling between those of the other two types of cell death. We believe that HT has a high potential for applications in the biological field for cellular 3D and internal structures tomography imaging. The 3D RI tomogram provides not only the morphological information of cells but also allows for quantitative analysis of physical parameters. With sufficient data collection, this information could be applicable for deep/machine learning to identify differences.

## Data Availability

The dataset underlying the results presented in this paper is not publicly available at this time but may be obtained from the authors upon reasonable request.
